# Quantum state transfer via Bloch oscillations

**DOI:** 10.1038/srep26054

**Published:** 2016-05-18

**Authors:** Dario Tamascelli, Stefano Olivares, Stefano Rossotti, Roberto Osellame, Matteo G. A. Paris

**Affiliations:** 1 Dipartimento di Fisica, Università degli Studi di Milano, I-20133 Milano, Italy; 2 Institut für Theoretische Physik & IQST, Albert-Einstein-Allee 11, Universität Ulm, Germany; 3 CNISM UdR Milano Statale, I-20133 Milano, Italy; 4 Istituto di Fotonica e Nanotecnologie, Consiglio Nazionale delle Ricerche, Piazza Leonardo da Vinci 32, I-20133 Milano, Italy; 5 Dipartimento di Fisica, Politecnico di Milano, Piazza Leonardo da Vinci 32, I-20133 Milano, Italy

## Abstract

The realization of reliable quantum channels, able to transfer a quantum state with high *fidelity*, is a fundamental step in the construction of scalable quantum devices. In this paper we describe a transmission scheme based on the genuinely quantum effect known as Bloch oscillations. The proposed protocol makes it possible to carry a quantum state over different distances with a minimal engineering of the transmission medium and can be implemented and verified on current quantum technology hardware.

The possibility of transferring, or sharing, a quantum state between different parties of a quantum network is of fundamental importance in quantum computation and communications systems. In solid-state implementations of quantum devices, for example, several small units need to be connected in order to share information among them, much the same way current (classical) computers components are. The realization of reliable channels, able to the transfer of quantum information with high *fidelity*, is therefore a fundamental step in the construction of a scalable quantum computer^1^. The latter, in turn, hold the promise of speeding up the solution of certain problems perceived as difficult on a classical computer^2^ and of enabling controlled simulations of the behavior of complex quantum systems^3^,^4^. Different quantum state transfer (QST) schemes have been proposed in the last decade. The range of systems that can be engineered for the task is quite large^5^,^6^. However, on ground of physical implementability and scalability, protocols that: i) avoid interactions with the system except at initialization and read-out; ii) are time-independent Hamiltonian, are to be preferred^6^. A periodical switching of a control field can be also of interest in view of an almost dispersionless transport over long distances^2^,^7^. Recently, an experimental verification on waveguide lattices^8^ of the perfect-state transfer protocols proposed in^9^,^10^,^11^ and^12^,^13^ has been reported in^14^,^15^ and^16^.

Here we propose a protocol to exploit Bloch oscillations^17^ in order to achieve nearly optimal state transfer. We use the probability of transfer of an information carrier between two different regions of a transmission line as a figure of merit and study the trade-off between the amount of resources used to prepare the initial state of the carrier and the transfer probability.

Our protocol requires a minimal engineering of the channel, consisting in the introduction of an externally tunable temperature^18^ gradient, or an electric field^19^. It offers the remarkable possibility of changing the transmission distance without modifying the geometry of the device. This feature represents a major innovation compared to previous QST protocols. The existing QST schemes are static: a given device, or *channel*, is able to transfer information only between two fixed endpoints. Our proposal, though based on a time-independent Hamiltonian, opens instead the possibility of dynamically reconfiguring the “routing” of the transmitted quantum information, a fundamental requirement in any quantum information processing device. This innovative scheme can be implemented and verified on current photonic lattices technology^18^,^19^,^20^,^21^ and can lead to the realization of the first reconfigurable QST device for photonic qubits. Moreover, it can find applications in all-optical switching of light in communication systems^22^.

## Results

### The model

The system we deal with is a 1D lattice. We indicate each site of the lattice by |*n*〉, 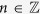. The system Hamiltonian is given by (we set 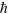 = 1):


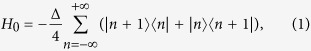


where Δ is the coupling between next neighbor sites. The same Hamiltonian also governs the dynamics of the Tight Binding Model (TBM)^23^ and of waveguide-array systems^8^. The corresponding Schrödinger equation is easily solved once we consider the representation in Bloch waves^24^,^25^, namely:


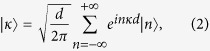


where *d* is the distance between each site composing the chain, with the (quasi-)momentum *κ* confined to the Brillouin zone −*π*/*d* ≤ *κ* ≤ +*π*/*d*. These states are eigenstates for the Hamiltonian (1) with eigenvalues


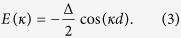


Equation (3) expresses the well-known momentum/energy dispersion relation in lattices.

**Figure 1:**
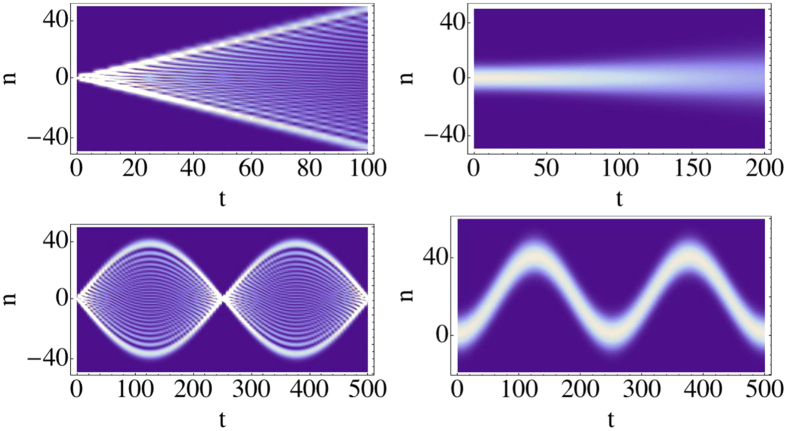
Evolution of the probability distribution |〈*n*|*ψ*(*t*)〉|^2^ (density plot) for Δ = 1, *F* = −1/40, *β* = 0.01 and for different initial conditions and system Hamiltonians. (Top left): Sharp initial condition 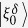 (4), *H* = *H*_0_ (1); (top right): Gaussian distribution 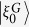 (5), *H* = *H*_0_; (bottom left): Sharp condition 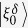, *H* = *H*_*B*_ (6); (bottom right): Gaussian wavepacket .., *H* = *H*_*B*_.

The evolution of a generic state 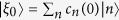 is obtained by the propagator *U*(*t*) = exp(−*iH*_0_*t*). In this simple setting we have 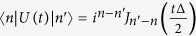, where *J*_*n*_(*x*) is the *n*-th Bessel-J function of the first kind. In Fig. 1 we show the evolution of two different initial conditions: a (sharp) localized condition (upper left frame) and a Gaussian wavepacket (upper right frame), both centered in the site labelled by 0, namely:


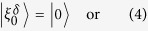



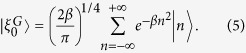


If our task is to transfer the excitation/electron/photon from the initial position *n*_*I*_ = 0 to a final position, or *target site*, *p* (*p* = 40 in the examples shown in the figures), this simple setup is completely uneffective. When starting from the sharp initial condition (4) the probability of reaching the target site decreases polynomially with the distance |*p*|. Starting from (5) leads to a diffusive behavior, since the initial momentum has mean 0 and variance 2*β*^24^.

### Bloch oscillations

We now add a linear potential to the Hamiltonian (1), mimicking the action of a “force” trying to pull the excitation in the desired direction. This could induce inter-band transitions^26^,^27^; however, since we are going to consider initial states with negligible transverse moment and small values of the force parameter, transitions to higher bands can be safely neglected^28^. We can therefore introduce the following single-band Hamiltonian:


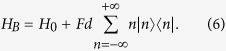


The eigenvalues of *H*_*B*_ are 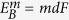 and the corresponding eigenstates are the Wannier-Stark states 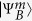, *m* = 0, ±1, …, where


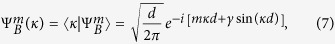


and *γ* = Δ/(2*dF*)^24^. The propagator, in the Bloch basis (2), is


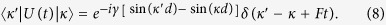


The quasi-momentum *κ* is changed by the *force* as *κ*(*t*) = *κ*(0) − *Ft*. On the other side, the group velocity *v*_*g*_(*κ*) of the wave, is defined through the dispersion relation (3) by


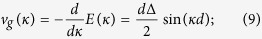


it changes sign every time the quasi-momentum *κ* reaches the boundaries of the first Brillouin zone, leading to Bloch oscillations^17^. The evolution of the initial states (4) and (5) in the presence of a potential is illustrated in Fig. 1. The appearance of the *breathing modes* (see lower left panel) is a consequence of the flat momentum spectrum corresponding to the sharp initial condition (4). The components having absolute initial momentum |*κ*| ≈ 0(±*π*) travel the furthest along the chain, reaching a distance Δ/*F*. Their speed, initially close to *d*Δ/2 sin(0(±*π*)) = 0, increases in module until it reaches the maximum value *d*Δ/2 at *t* = *π*/2*dF*. Then the velocity decreases in modulus until *t* = *π*/*dF* when it changes sign (Bragg reflection). The initially fastest components of the wave packet, corresponding to |*κ*| ≈ *π*/2, get Bragg reflected sooner, and are confined in a region (−Δ/2*F*, Δ/2*F*). The presence evenly distributed initial positive and negative momenta, leads moreover to an even spreading of the wavepacket over both the positive and negative axes; this leads to a further halving of the probability of reaching a target site *p*.

On the other side, if we take a Gaussian initial condition of the form (5), with 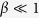, the distribution of the momentum can be peaked around *κ* = 0. The wavepacket will now travel in a definite direction, set by the sign of *F* and the shape of the starting Gaussian is preserved during the evolution. In fact, besides a phase factor 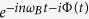
24 the coefficients distribution is the same as in (5) with the substitution: 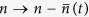, where 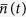 is the mean of the position observable 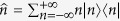. The second relevant point is that the center 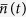 of this Gaussian shape performs an oscillation with period *T*_*B*_ = 2*π*/(*Fd*) within the coordinate space. So, as long as the initial shape is weakly localized in the coordinate space, we expect that the whole Gaussian shape - representing the probability distribution for each site - performs an oscillation with amplitude 2|*γ*|. We point out, moreover, that after a half period the coefficients *c*_*n*_(*t*) take the form:


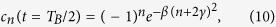


i.e. the wavefunction is the same as the initial one but shifted of −2*γ* and with alternate phase factors. Then, in this toy model, we are able to transfer the excitation from a site to another arbitrarily just varying the force acting on the system.

### Constraining the resources

**Figure 2:**
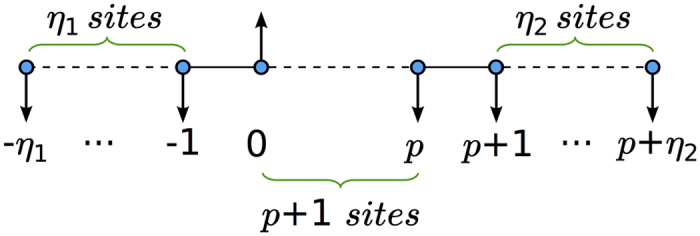
Finite chain representation.

We now want to understand under which conditions the dynamics on a finite chain approximates properly the one discussed so far. This issue is quite relevant since in any realistic setting the number of lattice sites or waveguides would be limited. Let *p* once more indicate the target site and suppose that the excitation is initially localized in a neighborhood of the site labelled by 0. We suppose to attach *η*_1_ sites before the site labelled by 0 and *η*_2_ after the one labelled by *p*, as shown in Fig. 2.

The total number of sites composing the chain is therefore *c* = (*p* + 1) + *η*_1_ + *η*_2_. In order so simplify the notation we introduce the quantities: *l* ≡ −*η*_1_ and *r* ≡ *p* + *η*_2_. Dealing with the finite case the Hamiltonian governing the system will be:


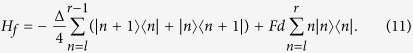


Of course, the Gaussian superposition in (5) cannot be extended to an infinite number of sites, but at most to the ones composing the chain. Moreover the state |*p*〉 must not appear in the Gaussian superposition: if it were the case, we would have a non-vanishing initial probability of finding the spin-excitation in the target site. It is clear then the necessity of taking the Gaussian superposition truncated in certain interval on the chain, so that it involves only a restricted number of sites. In particular we considered a Gaussian superposition symmetrically truncated with respect to its center. Chosen a truncation parameter *δ* < *η*_1_ we set the initial state to be:


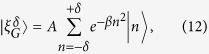


where *A* is a normalization factor, and investigate how the truncation affects the transport property. As a figure of merit we employ the probability of finding the excitation in a neighborhood of the final site *p*. Upon denoting by


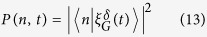


the probability of finding the spin excitation in the *n*-th site, we define the *success probability* as:


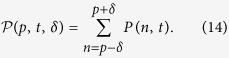


**Figure 3:**
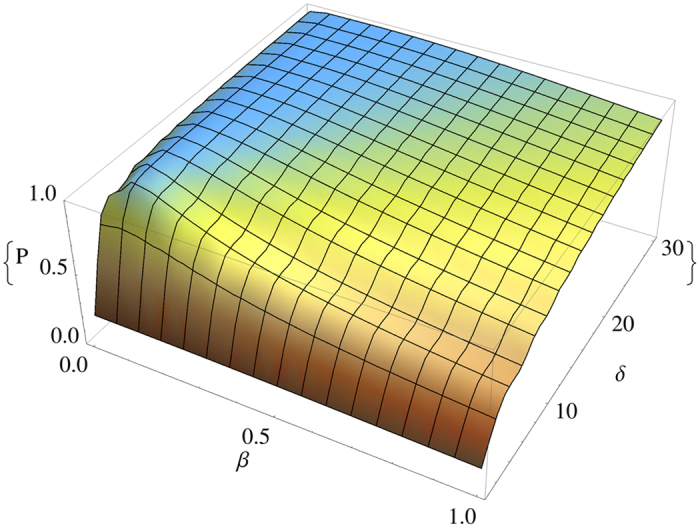
The success probability 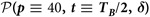 as a function of *β* and *δ* for Δ/*F* = −60.

Our main aim is to maximize such probability. The parameters we can control are the force intensity *F*, the truncation parameter *δ* and the Gaussian superposition width *β*. The value *F* is automatically set once we decide how far the excitation has to travel. Notice that, if *δ* is taken too close to 1 the Gaussian superposition becomes very similar to the sharp condition, leading to the unproductive breathing modes shown in the lower left panel of Fig. 1. As already discussed above, moreover, we need 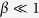 in order to have a momentum distribution peaked at 0. In our numerical simulations we set *η*_1_ = *η*_2_ = 2*δ* to avoid dangerous edge effects on the evolution of the truncated Gaussian superposition. As an example we plotted the values of *success probability* for Δ/*F* = −60 in Fig. 3 at the optimal time *t* = *T*_*B*_/2 (which does not depend on *β* or *δ*).

We notice that the mutual dependence of *β* and *δ* is clearly visible in all the region plotted. The behavior of the success probability at the vary of *δ* is clearly expected. In fact, as *δ* increases the region on which we collect the squared amplitudes *c*_*n*_(*T*_*B*_/2) covers an always wider part of the whole lattice. Obviously in the extreme case in which the region covered is the whole lattice the success probability is exactly 1. It is clear that exists a value for *β* for which the success probability is almost independent on *δ* and very close to 1. The value we find is about *β* = 0.01. For *δ* = 5 the success probability is already around 0.9 and reaches the value 1 for *δ* = 16. As we deal with a finite chain, it makes sense to take the lowest value of *δ* that makes the success probability larger than an assigned threshold value.

## Discussion

**Figure 4:**
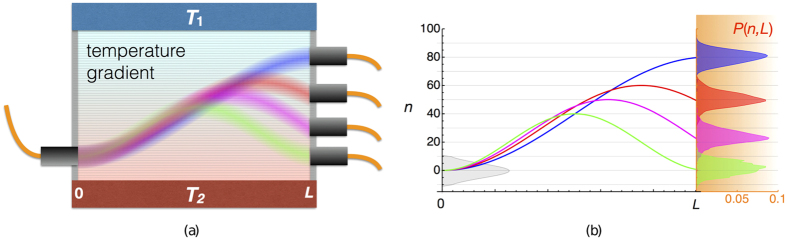
(**a**) A possible scheme of the experimental implementation with a simulation of the propagating signals from left to right. We set the coupling constant *J* = 1 and the initial condition is a truncated Gaussian with *β* = 0.01, *δ* = 10 centered at *n* = 0. The colors correspond to different values of *F*, determined by the temperature gradient between *T*_1_ and *T*_2_: *F* = 1/80 (blue), *F* = 1/60 (red), *F* = 1/50 (magenta), and *F* = 1/40 (green). (**b**) The left plot shows the mean position of the wavepackets simulated in the scheme (a) as a function of the distance from the input point; *n* refers to the number of the waveguide. On the right we show the intensity profiles *P*(*n*, *L*) of the wavepackets at the output of the waveguide array, as obtained through numerical simulations. The grey profile in the left plot refers to the the intensity profile of input truncated Gaussian where we used the same scale as for *P*(*n*, *L*) (not shown for the sake of clarity).

Now that we know in good approximation the dynamics of an excitation on a finite chain we can use the results obtained to perform an information transfer. The Hamiltonian (1), with *n* = 1, 2, …, *N* is equivalent to the one governing the propagation of the light in an array of *N* evanescently coupled optical waveguides^8^. The introduction of a linear potential will lead to solutions of the form (7), i.e. the Wannier-Stark states^19^,^24^. The external force acting on the lattice would be implemented by a linear gradient in the effective refractive index of the waveguides. This could be realized statically, but also dynamically, e.g. imposing a temperature gradient in the substrate and exploiting the thermo-optic effect^18^. In Fig. 4(a) we show the scheme of a possible implementation and the simulation of the propagating signals. We plot in Fig. 4(b) the mean position of the wavepackets simulated in (a) during the propagation through the waveguide array together with the intensity profiles *P*(*n*, *L*), defined as in (13), at the output: it is evident that the output states are well distinguishable and only slightly deformed with respect to the input [the grey profile in Fig. 4(b)]. In both figures the time parameter *t* has been replaced by the length *L* of the waveguide array.

In this setting, we have also an additional degree of freedom, namely the polarization of the light propagating in the array. This additional two-level degree of freedom does not interfere with the motion of the light in the lattice, as long as the coupling between the guides is polarization independent. We can then suppose that the light propagating through the lattice preserves its polarization. Light is thus carrying a qubit of information, encoded in its polarization state, from an initial region of the lattice to the target one. By modulating the external force the qubit could be displaced and dynamically redirected in different regions of the lattice.

The polarization state is analogous to a spin-1/2 state, characterized by the eigenstates |↓_*p*_〉 and |↑_*p*_〉, with respect to the Pauli operator 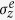. The necessary Hilbert space for such a degree of freedom is 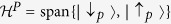. The overall Hilbert space needed for the complete description of the system is: 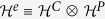. As the initial wavefuction we use 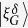 defined above with the addition of the polarization state, i.e.


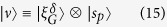


where 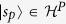 is the polarization state. Since in non-birefringent media the polarization of the light is not affected during the propagation, the Hamiltonian governing the motion of the electron through the lattice is 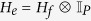, where 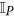 is the identity operator on the Hilbert space 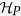. Under the influence of this Hamiltonian the light particle can carry quantum information under the form of its polarization from a site to another following the dynamics discussed above.

Waveguide arrays supporting such a dynamics could be fabricated by femtosecond laser writing^29^. This technique allows to directly inscribe high quality waveguides in glass substrates, exploiting the non-linear absorption of ultrashort laser pulses. This technology has widely proved its capabilities in producing complex three-dimensional waveguide arrays, able to reliably implement or simulate diverse quantum dynamics^30^,^31^ and, in particular, the Bloch oscillations of light^32^,^33^,^34^. Furthermore, it has been recently shown that femtosecond laser written circuits are specially suitable for the manipulation of polarization-encoded qubits, thanks to the relatively low birefringence that characterizes the waveguides fabricated with this technique^20^ and the possibility of fabricating polarization insensitive devices^35^.

Glass substrates are insensitive to external dc-generated fields^19^. To establish the required linear potential a stationary temperature gradient can be applied. In this case, the thermal gradient should stabilize before the state transfer begins. The response time is in the order of seconds, depending on the temperature gradient to be established, and it limits the switching rate that can be achieved. Finding a substrate that allows a much shorter reconfiguration time represents a technological challenge issued by the proposed protocol: it should have negligible birefringence and be sensitive to dc (or ac) fields at the same time.

A truncated Gaussian input state can be experimentally implemented in free space by using hard apertures together with a cylindrical telescope and a microscope objective to launch light in the array^36^. Although this method may be extremely effective to characterize the device and to demonstrate the quantum transfer effect, it may not be the best choice when this device will be used in an integrated environment, e.g. inside a quantum computer. In that case, it would be more appropriate to exploit engineered photonic lattices to transform the single mode of an incoming waveguide into a truncated Gaussian state that will constitute the input state of the quantum transfer device. In particular, the engineered photonic lattice could consist in a linear array of waveguides implementing a discrete fractional Fourier transform, as recently demonstrated by femtosecond laser waveguide writing in glass^37^. In such waveguide array, single waveguide excitation can produce a Gaussian output distribution. By connecting only a truncated set of such waveguides to the quantum transfer chip one would achieve the desired input state. The collection of the output state after the quantum transfer chip can be performed in two ways. The first one considers the case where just detection of the photons is required; in this case an array of multimode fibers, connected to the detectors, can be butt-coupled to the chip, where the core of each multimode fiber is large enough and of sufficiently high numerical aperture to collect the whole Gaussian distribution, as represented in Fig. 4(a). The second collection scheme considers instead the case when further processing of the signal is foreseen; in this case a coherent reduction of the Gaussian wavepacket to a single waveguide should be achieved. This task could be accomplished by using again a waveguide array that implements the discrete fractional Fourier transform and by taking into account the phases acquired in the transfer process. As described in (10), alternating phases will be present in the different output modes; such phases are however constant and fully predictable and can therefore be statically compensated by a suitable geometrical deformation of the waveguides^38^ before applying the Fourier transform module.

While Bloch oscillations are well known since the early stages of quantum mechanics, here we propose a way to take advantage of them for the task of quantum state transmission. We showed that the constraints imposed by the finiteness of the resources available for the preparation of the initial state induce a minor lowering of the protocol efficiency. The minimal amount of engineering required to implement the system Hamiltonian, and to prepare the initial state, make the realization of the protocol feasible with current quantum technology. Our results pave the way for further investigations concerning the system, such as the effects of noise^39^,^40^ and imperfections^41^ on the transmission probability.

## Additional Information

**How to cite this article**: Tamascelli, D. *et al.* Quantum state transfer via Bloch oscillations. *Sci. Rep.* **6**, 26054; doi: 10.1038/srep26054 (2016).
